# The Pin 1 inhibitor juglone attenuates kidney fibrogenesis via Pin 1-independent mechanisms in the unilateral ureteral occlusion model

**DOI:** 10.1186/1755-1536-3-1

**Published:** 2010-01-04

**Authors:** Shannon Reese, Aparna Vidyasagar, Lynn Jacobson, Zeki Acun, Stephane Esnault, Debra Hullett, James S Malter, Arjang Djamali

**Affiliations:** 1 Departments of Medicine and Surgery, University of Wisconsin-Madison School of Medicine and Public Health, Madison, WI, USA; 2 Department of Pathology and Laboratory Medicine, University of Wisconsin-Madison School of Medicine and Public Health, Madison, WI, USA

## Abstract

**Background:**

Pin 1 is a peptidyl-prolyl isomerase inhibitor related to cyclophilin A and FK506 binding protein (FKBP). Juglone (5-hydroxy-1,4-naphthoquinone) is a natural inhibitor of Pin 1 with anti-inflammatory and antifibrotic properties. We evaluated the role of Pin 1 in renal fibrogenesis by evaluating the effects of juglone on epithelial to mesenchymal transition (EMT) and fibrogenesis in the rat unilateral ureteral obstruction (UUO) model and normal rat tubular epithelial cells (NRK52E).

**Results:**

After 2 weeks of UUO, immunoblot analyses demonstrated that juglone (0.25 and 1 mg/kg/24 h) inhibited the deposition of matrix (α-smooth muscle actin (SMA), collagen type III and vimentin) and the activation of signaling pathways involved in fibrogenesis (phospho-smad2) and stress response (phospho-heat shock protein (HSP)27). Juglone also reduced EMT (α-SMA and E-cadherin dual staining) and oxidative stress (Mn superoxide dismutase (SOD) and NAPDH oxidase 2 (Nox-2) dual staining) in the obstructed kidney. There was no difference in Pin 1 levels between treatment and control groups. Pin 1 activity was significantly decreased in obstructed kidneys regardless of treatment status. *In vitro*, juglone (1 μM) significantly decreased α-SMA and p-smad levels compared to vehicle.

**Conclusions:**

Juglone attenuates fibrogenesis via Pin 1-independent mechanisms in the UUO model. The antifibrotic effects of juglone may result from the inhibition of smad2 and oxidative stress.

## Background

Obstructive nephropathy is a major cause of renal failure, particularly in infants and children [1,2]. Urinary tract obstruction and tubular dilatation result in a series of proinflammatory events that ultimately lead to chronic tubulointerstitial fibrosis and kidney failure [1,2]. Fibrogenesis starts with the activation of the renin-angiotensin system, tubular apoptosis and macrophage infiltration and is accompanied by the accumulation of interstitial fibroblasts from either proliferation of resident cells or epithelial to mesenchymal transition (EMT) [1,2]. The rodent unilateral ureteral obstruction (UUO) model has emerged as an important platform for the study of complex cellular interactions that regulate the development of interstitial inflammation, tubular apoptosis and interstitial fibrosis in this milieu [3]. Evidence suggests that the UUO model is reflective of human kidney disease [3]. Studies examining the mechanisms of fibrogenesis in UUO may therefore result in the development of therapies that will prevent or reverse the structural and functional consequences of obstructive nephropathy [3].

Pin 1 is a *cis-trans *peptidyl-prolyl isomerase (PPIase) related to cyclophilin A and FK506 binding protein (FKBP) [4,5]. Pin 1 modulates cytokine expression by activated T cells and eosinophils and participates in T cell and eosinophil apoptotic decisions both *in vitro *and *in vivo *[5]. In addition, Pin 1 blockade attenuates transforming growth factor β 1 (TGFβ 1) and granulocyte-macrophage colony-stimulating factor (GM-CSF) production and inflammation in experimental models of allergic lung fibrosis [4,6]. We therefore hypothesized that Pin 1 plays a role in kidney fibrogenesis and tested this hypothesis *in vivo *using the rodent UUO model and *in vitro *using normal rat proximal tubular epithelial cells (NRK52E). We used juglone (5-hydroxy-1,4-naphthoquinone) a natural inhibitor of Pin 1 to characterize the effects of Pin 1 inhibition on fibrogenesis.

## Results

### Juglone reduced fibrogenesis after UUO

**Figure 1:**
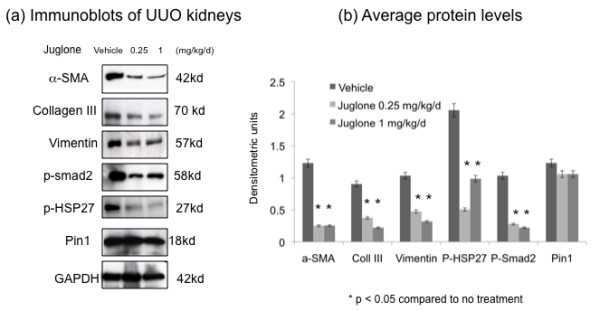
**Juglone reduced fibrogenesis after unilateral ureteral obstruction (UUO)**. Male Lewis rats (3 months old) underwent UUO of the left kidney for 2 weeks. There were three groups receiving vehicle or juglone (0.25 mg/kg/day or 1 mg/kg/day) for 2 weeks starting the day of surgery. **(a) **Immunoblot analyses of left kidney from control or juglone-treated rats for the proteins shown along the left. **(b) **Multiple (n = 3) immunoblots were quantitated and signals expressed as arbitrary units after normalization to glyceraldehyde 3-phosphate dehydrogenase (GAPDH) signal.

Male Lewis rats (3 months old) underwent UUO of the left kidney for 2 weeks. There were three groups receiving vehicle, juglone 0.25 mg/kg/day or juglone 1 mg/kg/day for 2 weeks starting the day of surgery. There was no animal death associated with treatment. Treated animals had a 10% weight loss in the first week after surgery, which resolved by the end of week 2. Immunoblot analyses for Pin 1, biomarkers of matrix remodeling (α-smooth muscle actin (SMA), collagen type III and vimentin) and signaling pathways involved in fibrogenesis (phospho-smad2) and stress response (phospho-heat shock protein (HSP)27) demonstrated that juglone therapy decreased α-SMA, collagen type III, vimentin, p-smad2 and p-HSP27 levels (Figure 1). There was no difference in Pin 1 levels between treatment and control groups suggesting that juglone inhibits fibrogenesis independently of Pin 1 levels in the UUO model.

### Juglone and UUO had similar inhibitory effects on Pin 1 activity

**Figure 2:**
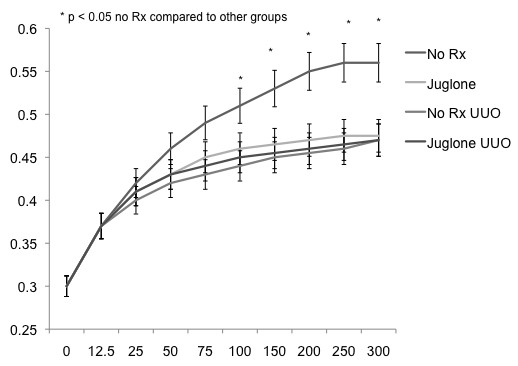
**Juglone and unilateral ureteral obstruction (UUO) had similar inhibitory effects on Pin 1 activity**. Pin 1 activity was measured in whole kidney protein lysates after 2 weeks of UUO as previously described [18]. Mean values and standard error bars from UUO and control kidneys are represented for each time point.

We next examined Pin 1 activity in unobstructed and obstructed kidneys in control or juglone-treated rats. These analyses would help us determine whether the antifibrotic properties of juglone resulted from Pin 1 blockade. The studies demonstrated that juglone effectively inhibited Pin 1 activity in unobstructed right kidneys (Figure 2). However, Pin 1 activity was significantly decreased in left obstructed kidneys regardless of treatment status (Figure 2). Pin 1 activity in obstructed kidneys was reduced to the same level as in unobstructed kidneys treated with juglone. In aggregate, these studies suggest that the antifibrotic effects of juglone are independent from Pin 1 blockade during UUO.

### Juglone reduced EMT and oxidative stress after UUO

**Figure 3:**
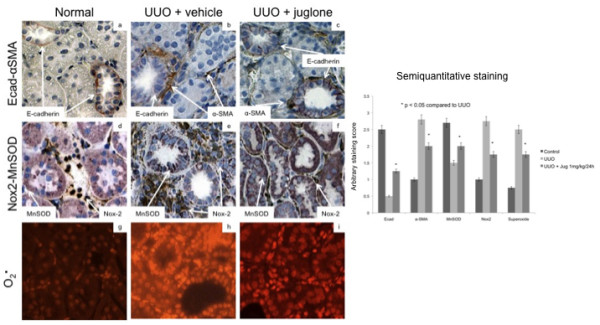
**Juglone reduced EMT and oxidative stress after unilateral ureteral obstruction (UUO)**. Right (control) and left (UUO) kidneys were removed after 2 weeks, fixed and stained with antibodies for E-cadherin (pink, epithelial marker) and α-smooth muscle actin (SMA) (brown, mesenchymal marker) to evaluate epithelial to mesenchymal transition (EMT), and Mn superoxide dismutase (MnSOD) (pink), NADPH odixase 2 (Nox-2) (brown) and superoxide (red fluorescent) to assess oxidative stress. Experimental conditions are shown along the top while antibody and dyes that were utilized are along the left side of the panels. Aggregate semiquantitative scores are presented in the right panel. Cortical staining intensity was scored on a scale of 0 to 3 (0 no staining, 1 mild, 2 moderate, 3 intense) for E-cadherin (distal tubules), α-SMA (interstitium), MnSOD (tubules), Nox-2 (interstitium) and superoxide (tubulointerstitium). Five high magnification fields were evaluated per kidney (five kidneys in each group) and results were expressed as mean and standard error bars.

To further define the effects of juglone on fibrogenesis, we assessed EMT using double-staining immunohistochemical analyses for E-cadherin (pink, epithelial marker) and α-SMA (brown, mesenchymal marker) in normal right and UUO left kidneys in control or juglone-treated rats (Figure 3a-c). These studies showed pink basolateral E-cadherin staining in distal tubules of control kidneys (Figure 3a). UUO resulted in downregulation of E-cadherin (no basolateral staining) and greater brown interstitial staining for α-SMA (Figure 3b). Treatment with juglone preserved distal E-cadherin expression and decreased interstitial α-SMA levels (Figure 3c), suggesting that juglone may attenuate EMT during UUO. Aggregate semiquantitative scoring is presented as a bar graph in the right panel (Figure 3).

We evaluated oxidative stress using dual-staining analyses for antioxidant (Mn superoxide dismutase (SOD)) and pro-oxidant (NAPDH oxidase 2 (Nox-2)) enzymes (Figure 3d-f). MnSOD is a superoxide scavenger while Nox-2 is one of the key generators of superoxide in the kidney [7]. Normal kidneys showed strong tubular MnSOD staining and rare patchy areas of monocytic infiltration positive for Nox-2 (Figure 3d). UUO resulted in reduced tubular MnSOD and increased tubulointerstitial Nox-2 (Figure 3e) consistent with significant monocyte/macrophage infiltration and a pro-oxidant milieu. Juglone attenuated these changes suggesting that treatment decreases oxidative stress (Figure 3f). Consistent with these findings, dihydroethidine staining for superoxide anion was significantly decreased with juglone compared to vehicle (Figure 3g, h). Aggregate semiquantitative scoring is presented as a bar graph in the right panel (Figure 3).

### The effects of juglone on fibrogenesis may be mediated by smad2

**Figure 4:**
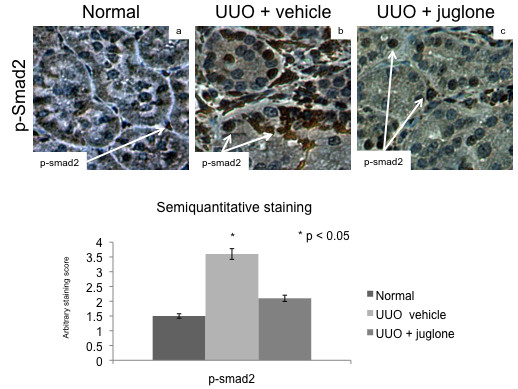
**Juglone reduced nuclear smad2 activity in unilateral ureteral obstruction (UUO)**. Right (control) and left (UUO) kidneys were fixed and stained with antibodies for phospho-smad2 (brown) to assess smad2 activity. Experimental conditions are shown along the top. Aggregate semiquantitative scores are presented in the right panel.

**Figure 5:**
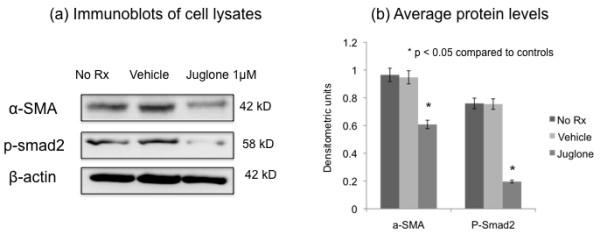
**The effects of juglone on fibrogenesis may be mediated by smad2. **Proximal tubular cells were untreated, treated with vehicle, or juglone as shown for 48 h prior to lysis and **(a) **western blotting for the proteins shown along the left. In **(b)**, three independent experiments were quantitated after normalization to actin signals.

To determine whether juglone inhibited TGFβ 1 activity we evaluated phospho-smad2 activity in normal kidneys compared to obstructed kidneys treated or not with juglone (Figure 4). These studies demonstrated that nuclear p-smad2 was significantly increased after UUO and that juglone prevented nuclear p-smad2 activity. Lastly, we evaluated the effects of juglone on α-SMA and activated smad2 (p-smad2) levels in proximal tubular epithelial cells. Juglone (1 μM) significantly reduced α-SMA and p-smad2 levels, consistent with our *in vivo *studies and suggesting that juglone may inhibit smad2 phosphorylation and activation in tubular epithelial cells (Figure 5).

## Discussion

In the present work we demonstrate that juglone, a naturally occurring Pin1 isomerase inhibitor, attenuates fibrogenesis in kidneys undergoing obstructive injury. This effect appears to be Pin 1 independent as PPIase activity was unchanged in the UUO left kidney between control and juglone-treated rats. Our studies further suggest that the antifibrotic effects of juglone result from the inhibition of smad2 phosphorylation and oxidative stress.

Juglone is a napthaquinone found in the leaves, roots and bark of plants from the walnut family. It is toxic to the growth of non-walnut plants and likely exerts its effect by inhibiting peptidyl-prolyl isomerases found in plants. Juglone has differential effects on cell cycling and metabolism depending on the species, organ and drug concentrations [8]. In F344 rats, high concentrations of juglone-derived radioactivity were found in the kidney after oral, intravenous and subcutaneous dosing [9]. The accumulation in the kidney was attributed to covalent binding of juglone and its metabolites to cytosolic proteins and suggests that the kidney may be a potential treatment target for juglone. In support of this hypothesis, juglone increased the activities of phase II detoxification enzymes quinone reductase and glutathione transferase in the kidney of Sprague-Dawley rats suggesting a role for this compound to protect animals against toxin-induced kidney injury [10].

Our results are in agreement with previous observations addressing the antifibrotic and anti-inflammatory characteristics of juglone in experimental models of lung injury [6,11]. These studies demonstrated that juglone therapy selectively inhibited eosinophilic and lymphocytic inflammation in rats undergoing experimental allergic lung fibrosis [6] and lung allograft rejection [11]. Juglone reduced eosinophilic pulmonary inflammation, TGFβ 1, collagen expression and airway remodeling in rats undergoing allergic lung fibrosis [6]. Similarly, juglone treatment prevented the acute and chronic rejection of major histocompatibility complex (MHC)-mismatched, orthotopic rat lung transplants by reducing the expression of proinflammatory interferon (IFN)γ and CXC chemokine ligand (CXCL)10 cytokines [11]. In these studies, combined transcriptional and post-transcriptional blockade of cytokine expression with cyclosporine A and the juglone was synergistic [11]. Our findings extend these data by demonstrating that juglone also attenuates inflammation in a macrophage-driven kidney injury model [1-3].

Interestingly, the anti-inflammatory effects of juglone were not dependent on Pin 1 blockade in the UUO model. Rather, they were associated with the inhibition of oxidative stress and smad2 phosphorylation. Juglone may have either pro or antioxidant characteristics depending on the milieu and drug concentrations [8]. While its use can result in the generation of reactive oxygen species in *Caenorhabditis elegans *[12], juglone is a potent antioxidant in human cortical neurons [13]. Juglone treatment prevents oxidative and heat stress-induced dephosphorylation of Tau (an important step in the pathogenesis of Alzheimer's disease) in primary brain cortical cultures [13]. Oxidative stress is a common injury pathway involved in kidney fibrogenesis [14,15]. We recently demonstrated that specific inhibitors of Nox (the primary generator of superoxide anion in the kidney) decreased fibrogenesis in kidney allografts by decreasing fibronectin and phospho-smad2 and increasing E-cadherin levels [7]. We have now demonstrated that juglone improves the oxidative stress balance in the UUO model by downregulating Nox-2 and superoxide anion while increasing tubular MnSOD levels. In addition, we have shown that juglone inhibits the phosphorylation of smad2 an important redox-sensitive, profibrotic signaling molecule in the kidney [7,16,17]. Although it is unclear whether the inhibition of smad2 phosphorylation was a direct effect or a downstream event secondary to juglone's antioxidant characteristics in our model, smad2 inhibition has been successful in experimental studies of native and transplants kidney fibrosis [7,16,17].

## Conclusions

In summary, these studies demonstrate that juglone attenuates fibrogenesis in kidneys undergoing obstructive injury via Pin 1-independent mechanisms. The antifibrotic effects of juglone may result from the inhibition of inflammation and more specifically smad2 and oxidative stress. Future studies are needed to determine the cellular and molecular mechanisms that regulate the inhibitory effects of juglone on smad and Nox molecules.

## Methods

### Animals

Adult (9 to 11 weeks old) male Lewis rats were purchased from Harlan Teklad (Madison, WI, USA). Animals were housed in the animal care facility at the William Middleton Veterans Affairs Hospital (VAH) in Madison, WI, USA, and the procedures were performed in accordance with the animal care policies at the VAH and the University of Wisconsin. The UUO procedure was performed under general anesthesia with isoflurane as described previously [14]. Briefly, the left ureter was ligated with 6-0 silk at two points and then severed between the ligatures to prevent retrograde urinary tract infection. Control animals underwent surgery and received vehicle (10% ethanol) (n = 5). Juglone was administered intraperitoneally for 14 days at 0.25 (n = 5) and 1 mg/kg/24 h diluted in 1 ml of vehicle (n = 5). Animals were killed after 2 weeks by exsanguination through cardiac puncture under general anesthesia. Both kidneys were harvested and sectioned longitudinally in half. Half was snap frozen immediately and used for immunoblot analysis and the other half was formalin fixed and paraffin embedded for immunohistochemical analyses. The right kidney served as the control to the left obstructed kidney.

### Immunoblotting

Western blotting was performed on protein lysates obtained from whole kidney tissue or cell lysates as described previously [14]. Briefly, after separation by SDS-PAGE (10% to 20% gradient PAGE, Bio-Rad, Hercules, CA, USA) proteins were transferred electrophoretically (100 V, 30 min) to nitrocellulose membranes (Bio-Rad) that were then blocked with a solution containing 5% non-fat milk, 50 mM Tris, HCl, pH 7.4, NaCl 150 mM, Tween 20 0.05% (TBS-Tween) overnight at 4°C. Membranes were incubated the next day with antibodies against α-SMA (2,000^-1^), Vimentin (100^-1^), collagen type III (100^-1^), phospho-HSP27 (0.25^-1^), phospho-smad2 (2,500^-1^), Pin 1 (200^-1^), and GAPDH (1:5,000). Binding of primary antibodies was followed by incubation for 1 h at room temperature with a secondary horseradish peroxidase (HRP)-conjugated IgG in 1% non-fat milk. Signals were visualized by enhanced chemiluminescence signals captured on x-ray films. Data was normalized to GAPDH. Densitometry was performed using the NIH Image J software http://rsbweb.nih.gov/ij/.

### Immunohistochemical analyses

A portion of the kidney tissue was excised promptly after the animals were killed. It was immediately placed in 10% neutral-buffered formalin. Tissue was fixed overnight in formalin and processed for paraffin embedding following standard protocols and then sectioned for antibody staining. Double staining of E-cadherin/α-SMA and Nox-2/MnSOD was performed after sections were deparaffinized and hydrated. Heat-induced antigen retrieval was performed using a 5 mM ethylenediaminetetra-acetic acid (EDTA) solution (pH = 8.0) and a 10 mM citrate solution (pH = 6.0), respectively, at 25 psi for 2 min in a decloaking chamber. Non-specific staining was blocked using Sniper (Biocare Medical, Concord, CA, USA) for 9 min. Slides were incubated overnight with p-smad2 (500^-1^), E-cadherin (50^-1^) or Nox-2 (50^-1^) then washed and incubated with 3% hydrogen peroxide for 30 min. MACH 2 HRP polymer detection system (Biocare Medical, Concord, CA, USA) and 3,3'-diaminobenzidine (DAB) substrate were used to tag and stain the first primary antibody brown. Slides were then incubated with the second primary antibody α-SMA (50,000^-1^) or MnSOD (5,000^-1^) at room temp for 1 h. MACH 2 HRP polymer detection system and VIP substrate (Vector Laboratories, Burlingame, CA, USA) were used to tag and stain the second primary antibody purple. Tissue sections were washed in distilled water, counterstained with hematoxylin, dehydrated through an ethanol series and mounted with cover slips. Harris hematoxylin and 1% alcohol eosin were used to assess overall kidney injury and morphology. A total of 25 μM dihydroethidine dye (Molecular Probes, Carlsbad, CA, USA) was used for superoxide anion staining according to the manufacturer's recommendations.

Cortical staining intensity was scored on a scale of 0 to 3 (0 no staining, 1 mild, 2 moderate, 3 intense) for E-cadherin (distal tubules), α-SMA (interstitium), MnSOD (tubules), Nox-2 (interstitium), p-smad2 and superoxide (tubulointerstitium). Five high magnification fields were evaluated per kidney and results were expressed as mean and standard error bars.

### Pin 1 activity assay

Pin 1 activity was measured in whole kidney protein lysates as described previously [18]. Briefly, tissue lysates were prepared by five freeze-thaw cycles in a buffer containing 50 mM 4-(2-hydroxyethyl)-1-piperazineethanesulfonic acid (HEPES) and 100 mM NaCl (pH 7.0). Total protein (10 μg) in 10 μl was mixed with 70 μl of the HEPES/NaCl buffer supplemented with 2 mM dithiothreitol (DTT) and 0.04 mg/ml bovine serum albumin (BSA). Then, 5 μl of chymotrypsin (60 mg/ml in 0.001 N HCl) was added and thoroughly mixed. Finally, 5 μl of the substrate Suc-AEPF-pNa (provided by Peptides International, Louisville, KY, USA) dissolved in dimethyl sulfoxide (DMSO) and prepared at 100 μg/ml in 480 mM LiCl/trifluoroethanol was added. The absorption at 390 nM, which detects the formation of free p-nitroanilide (pNA), was monitored using a Beckman Coulter

DU 800 spectrophotometer (Brea, CA, USA). All of the reagents and materials were kept at 4°C during the procedure. Mean values and standard error bars from UUO and control kidneys are represented for each time point.

### Juglone *in vitro *experiments

Normal rat kidney proximal epithelial cells (NRK52E) were obtained from the American Type Culture Collection (ATCC, Rockwell, MD, USA) and maintained at 37°C in a humidified atmosphere containing 5% CO_2_. Cells were seeded at 2.5 × 10^5 ^cells per well into six-well culture plates in Dulbecco modified Eagle medium (DMEM; high glucose) containing 5% heat inactivated fetal bovine serum (FBS), 44 mM NaHCO_3_, 5,000 IU penicillin and 5,000 μg/ml streptomycin (Cellgro, VA, USA). At 80% confluency, media was changed to serum free DMEM supplemented with 0.1% BSA for 12 h to arrest growth and synchronize cell activity. Cells were treated with juglone (1 μM) or vehicle (0.01% ethanol) for 48 h. Studies were performed in triplicates. Western blots for α-SMA, phospho-smad2 and β-actin were performed as described above.

### Statistical analysis

The Student *t *test and the non-parametric Mann-Whitney rank sum test (Sigma Stat Software, Jandel Scientific, Chicago, IL, USA) were utilized when appropriate to compare differences in Pin 1 activity and gene and protein expression between groups. *P *values ≤ 0.05 were considered significant.

## Competing interests

The authors declare that they have no competing interests.

## Authors' contributions

AD and JSM developed the concept and design of the study. SR, AV, LJ, ZA and SE contributed to data acquisition and analyses. SR, SE, DH, JM and AD contributed to the writing of the manuscript and all the authors approved the final manuscript.

## References

[B1] Bascands JL Schanstra JP Obstructive nephropathy: Insights from genetically engineered animals Kidney Int 2005 68 925 937 10.1111/j.1523-1755.2005.00486.x 16105023 PMC1885837

[B2] Chevalier RL Obstructive nephropathy: towards biomarker discovery and gene therapy Nat Clin Pract Nephrol 2006 2 157 168 10.1038/ncpneph0098 16932414

[B3] Klahr S Morrissey J Obstructive nephropathy and renal fibrosis Am J Physiol Renal Physiol 2002 283 F861 F875 12372761 10.1152/ajprenal.00362.2001

[B4] Esnault S Rosenthal LA Shen ZJ Sedgwick JB Szakaly RJ Sorkness RL Malter JS A critical role for Pin1 in allergic pulmonary eosinophilia in rats J Allergy Clin Immunol 2007 120 1082 1088 10.1016/j.jaci.2007.06.024 17720236

[B5] Esnault S Shen ZJ Malter JS Pinning down signaling in the immune system: the role of the peptidyl-prolyl isomerase Pin1 in immune cell function Crit Rev Immunol 2008 28 45 60 18298383 10.1615/critrevimmunol.v28.i1.30

[B6] Shen ZJ Esnault S Rosenthal LA Szakaly RJ Sorkness RL Westmark PR Sandor M Malter JS Pin1 regulates TGF-beta1 production by activated human and murine eosinophils and contributes to allergic lung fibrosis J Clin Invest 2008 118 479 490 10.1172/JCI34527 18188456 PMC2176187

[B7] Djamali A Vidyasagar A Adulla M Hullett D Reese S Nox-2 is a modulator of fibrogenesis in kidney allografts Am J Transplant 2009 9 74 82 10.1111/j.1600-6143.2008.02463.x 18976289 PMC3572864

[B8] Chobot V Hadacek F Milieu-dependent pro- and antioxidant activity of juglone may explain linear and nonlinear effects on seedling development J Chem Ecol 2009 35 383 390 10.1007/s10886-009-9609-5 19263168

[B9] Chen LJ Lebetkin EH Burka LT Metabolism and disposition of juglone in male F344 rats Xenobiotica 2005 35 1019 1034 10.1080/00498250500356621 16393859

[B10] Munday R Munday CM Induction of quinone reductase and glutathione transferase in rat tissues by juglone and plumbagin Planta Med 2000 66 399 402 10.1055/s-2000-8576 10909256

[B11] Esnault S Braun RK Shen ZJ Xiang Z Heninger E Love RB Sandor M Malter JS Pin1 modulates the type 1 immune response PLoS ONE 2007 2 e226 10.1371/journal.pone.0000226 17311089 PMC1790862

[B12] de Castro E Hegi de Castro S Johnson TE Isolation of long-lived mutants in Caenorhabditis elegans using selection for resistance to juglone Free Radic Biol Med 2004 37 139 145 10.1016/j.freeradbiomed.2004.04.021 15203185

[B13] Galas MC Dourlen P Bégard S Ando K Blum D Hamdane M Buée L The peptidylprolyl cis/trans-isomerase Pin1 modulates stress-induced dephosphorylation of Tau in neurons. Implication in a pathological mechanism related to Alzheimer disease J Biol Chem 2006 281 19296 19304 10.1074/jbc.M601849200 16675464

[B14] Vidyasagar A Reese S Acun Z Hullett D Djamali A HSP27 is involved in the pathogenesis of kidney tubulointerstitial fibrosis Am J Physiol Renal Physiol 2008 295 F707 716 10.1152/ajprenal.90240.2008 18596079 PMC2536879

[B15] Djamali A Oxidative stress as a common pathway to chronic tubulointerstitial injury in kidney allografts Am J Physiol Renal Physiol 2007 293 F445 455 10.1152/ajprenal.00037.2007 17459952

[B16] Li JH Zhu HJ Huang XR Lai KN Johnson RJ Lan HY Smad7 inhibits fibrotic effect of TGF-beta on renal tubular epithelial cells by blocking Smad2 activation J Am Soc Nephrol 2002 13 1464 1472 10.1097/01.ASN.0000014252.37680.E4 12039975

[B17] Rhyu DY Yang Y Ha H Lee GT Song JS Uh ST Lee HB Role of reactive oxygen species in TGF-beta1-induced mitogen-activated protein kinase activation and epithelial-mesenchymal transition in renal tubular epithelial cells J Am Soc Nephrol 2005 16 667 675 10.1681/ASN.2004050425 15677311

[B18] Esnault S Shen ZJ Whitesel E Malter JS The peptidyl-prolyl isomerase Pin1 regulates granulocyte-macrophage colony-stimulating factor mRNA stability in T lymphocytes J Immunol 2006 177 6999 7006 17082615 10.4049/jimmunol.177.10.6999

